# Association between Apolipoprotein E genotype and functional outcome in acute ischemic stroke

**DOI:** 10.18632/aging.204460

**Published:** 2023-01-13

**Authors:** Xiaoming Rong, Jingjuan Chen, Dong Pan, YuKai Wang, Chengguo Zhang, Yamei Tang

**Affiliations:** 1 Department of Neurology, Sun Yat-sen Memorial Hospital, Sun Yat-sen University, Guangzhou, People’s Republic of China; 2 Department of Neurology, First People’s Hospital of Foshan, Foshan, People’s Republic of China; 3 Guangdong Provincial Key Laboratory of Malignant Tumor Epigenetics and Gene Regulation, Sun Yat-Sen Memorial Hospital, Sun Yat-Sen University, Guangzhou, People’s Republic of China; 4 Guangdong Province Key Laboratory of Brain Function and Disease, Zhongshan School of Medicine, Sun Yat-Sen University, Guangzhou, People’s Republic of China

**Keywords:** Apolipoprotein E, stroke, neutrophil-to-lymphocyte ratio, functional outcome

## Abstract

This study aims to determine whether APOE alleles would affect the functional outcome in acute ischemic stroke (AIS) and whether the relationship between inflammation and stroke-related disability varies according to APOE genotypes. We retrospectively collected the demographic and clinical data of AIS patients within one week of symptom-onset through medical records review. The primary outcome was dependence or death, defined as modified Rankin scale (mRS) score of 2–6, which was assessed at 3 months. Among 1929 enrolled patients, the prevalence of APOE ε4 carriers was 17.73% (342/1929). There were 394 AIS patients (394/1929, 20.43%) showed poor function outcome of 90-day mRS (2–6), of whom 147 (147/342, 42.98%) were APOE ε4 carriers and 247 (247/1587, 15.56%) were non-ε4 carriers. There was a significant increased probability of poor functional outcome after AIS among APOE ε4 carriers versus non-ε4 carriers (adjusted-OR 4.62, 95% CI 3.51 to 6.09, *P* < 0.001). Among ε4 carriers, high neutrophil-to-lymphocyte ratio (NLR) was significantly associated with stroke-related disability (P_trend_ = 0.035); however, no significant association was observed among non-ε4 carriers. Our study showed that the APOE ε4 carriers had worse functional outcome after AIS as compared with non-ε4 carriers. APOE genotype may modify the relationship between NLR and 3-month stroke outcome.

## INTRODUCTION

Stroke is the second highest cause of death globally and a major cause of disability worldwide [1]. Many controllable and uncontrollable factors are associated with stroke outcome. Patients with similar severity of symptoms and vascular conditions may have a different prognosis even if they receive standard treatment within the same time window. Even though the current guidelines have detailed recommendations for the early management [2], the prognosis for recovery varies, suggesting the impact of individualized uncontrollable factors on the prognosis of stroke.

Apolipoprotein E (APOE) has three major isoforms (APOE 2,3,4) which is encoded by three different alleles located on chromosome 19. It is abundantly expressed in multiple brain cell types, including astrocytes, microglia, neuron and vascular mural cells, and causes cell type-specific functions [3–5]. APOE isoforms impact cardiovascular, neurological and infectious diseases. APOE ε4 isoform is the strongest genetic risk factor for Alzheimer’s disease [6, 7], and it has neuropathological effects on neurons and the blood-brain barrier resulting in various clinical manifestations after brain injury [3, 4]. Previous studies reported that the possession of the APOE ε4 allele was associated with unfavorable outcome in chronic central nervous system disorders, including Alzheimer’s disease [7], Parkinson’s disease [8], amyotrophic lateral sclerosis [9, 10], as well as in acute brain injuries including intracerebral hemorrhage [11] and post stroke dementia [12]. However, for acute ischemic stroke (AIS), previous studies showed controversial results about the association between APOE polymorphism and clinical outcomes [13]. The reasons may be partly due to different target population, varied outcome time point and confounding factors. For example, Broderick’s study only focused on patients with acute ischemic stroke who were receiving intravenous tissue plasminogen activator [14]. McCarron’s study had a relatively small sample size of AIS [15]. Gromadzka’s study indicated the ε4 genotype as a significant independent positive predictor of poor outcome and its time frame was within 1 year after ischemic stroke [16].

Apart from lipo-protein metabolism, APOE may also modify other risk factors for vascular injury, such as inflammation. Although association between APOE ε4 genotype and unfavorable outcome have been reported in ischemic stroke [16], there are relatively limited data with adjustment for inflammation level. The neutrophil-to-lymphocyte ratio (NLR) is easily assessable and is a well-known marker of systemic inflammation and infection. Growing evidence proved that higher admission NLR increased the risk of poor outcome at 3-month in patients with AIS [17–21]. However, one single index may not be enough to capture the complexity of the immune status and inflammation response. The interaction of APOE genotype and NLR on ischemic stroke outcome has not been fully addressed.

To evaluate the relationship between APOE isoforms and stroke outcome and to investigate the potential interaction of APOE genotype and inflammation level in patients with AIS, we undertook this retrospective cohort study of AIS and follow-up for 3-month functional outcome. We excluded patients who had received intravenous tissue plasminogen activator and/or mechanical thrombectomy because these two strategies have great impact on the prognosis of stroke. We hypothesized that APOE ε4 allele may be a predictor for poor functional outcome in patients with AIS, furthermore, APOE ε4 carrier status may modify the previously established relationship between NLR and stroke outcome.

## RESULTS

**Figure 1:**
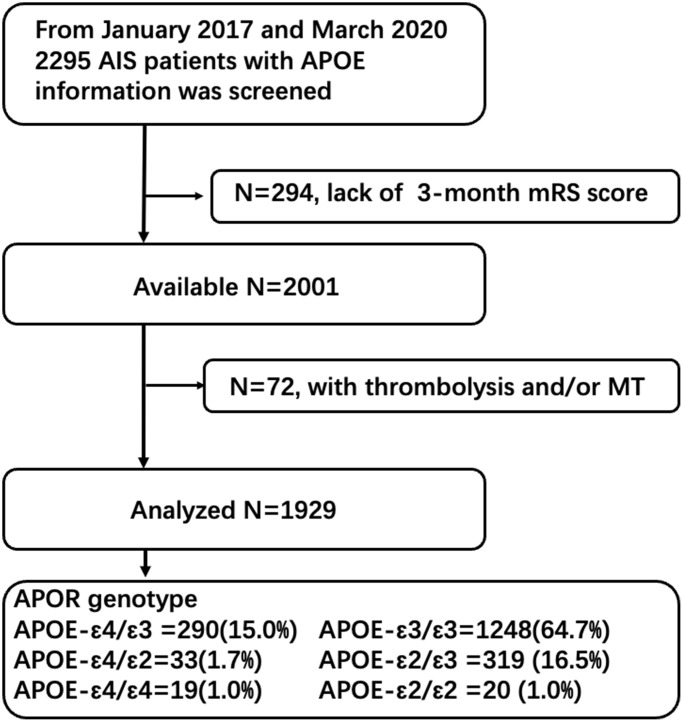
Study flowchart.

Among 2295 AIS patients with APOE genotype testing screening from 2017-2020, 2001 patients had 3-month follow-up information. We further excluded 72 patients with thrombolysis and or endovascular thrombectomy (Figure 1). After application of our exclusion criteria, 1929 patients (mean/sd age 65.32/12.21 years) were included in our study.

Among these patients, 1248 (64.7%) were APOE-ε3/ε3, 290 (15.0%) were ε4/ε3, 33(1.7%) were ε4/ε2 and 19 (1.0%) were homozygous (ε4/ε4). APOE ε4 carriers were less likely to have habit of smoking (21.6% vs. 27.1%, *p* = 0.04). However, APOE ε4 carriers and non-ε4 carriers had similar vascular risk factors (hypertension 66.67% vs. 70.83%, *p* = 0.13; diabetes 27.49% vs. 30.31%, *p* = 0.30) as well as stroke severity (NIHSS, *p* = 0.23) (Table 1, Supplementary Table 1). Considering APOE genotype may be related to lipid metabolism and systematic inflammation, we compared the lipid level, homocysteine, NLR and C-reactive protein (CRP) between APOE ε4 carriers and non-ε4 carriers. Table 1 showed that patients with ε4 had higher low-density lipoprotein (*p* < 0.001), total cholesterol (*p* = 0.002), triglyceride (*p* = 0.027), and small and low-density lipoprotein cholesterol (*p* < 0.001) as compared with non-ε4 carriers. In addition, ε4 carriers tend to have higher NLR (3.78 vs. 3.15, *p* < 0.001). However, ε4 carriers was found to have lower homocysteine level (11.85 vs. 12.70, *p* < 0.001).

**Table 1:** Baseline clinical characteristics of the study sample.

**Characteristic**	**APOE-ε4 genotype**			***P* value**
	**Entire cohort (*n* = 1929)**	**ε4 (*n* = 342)**	**Non ε4 (*n* = 1587)**	
Age, mean/SD years (*n* = 1929)	65.32/12.21	64.93/12.58	65.40/12.14	0.53
Male sex, *n* (%) (*n* = 1929)	1277 (66.20)	221 (64.62)	1056 (66.54)	0.50
Education >9 years, *n* (%) (*n* = 1929)	181 (9.38)	32 (9.36)	149 (9.39)	0.98
Atrial fabulation, *n* (%) (*n* = 1929)	141 (7.31)	17 (4.97)	124 (7.81)	0.07
Hypertension, *n* (%) (*n* = 1929)	1352 (70.09)	228 (66.67)	1124 (70.83)	0.13
Diabetes mellitus, *n* (%) (*n* = 1929)	575 (29.81)	94 (27.49)	481 (30.31)	0.30
Stroke history, *n* (%) (*n* = 1884)	76 (4.03)	15 (4.5)	61 (3.9)	0.31
Smoke, *n* (%) (*n* = 1929)	504 (26.13)	74 (21.64)	430 (27.10)	0.04
Drinking, *n* (%) (*n* = 1929)	301 (15.60)	48 (14.04)	253 (15.94)	0.38
NIHSS score, median (range) (*n* = 1929)	3 (0, 29)	3 (0, 25)	3 (0, 29)	0.23
HbAlc, median (range) (*n* = 1759)	5.9 (3.2, 15.9)	6.0 (4, 14.1)	5.9 (3.2, 15.9)	0.16
***Lipid level,*** median (range)				
Hdl, mmol/L (*n* = 1856)	1.1 (0.08, 3.32)	1.0 (0.57, 3.32)	1.1 (0.08, 2.76)	0.74
LDL, mmol/L (*n* = 1856)	2.51 (0.62, 7.94)	2.68 (0.64, 7.07)	2.46 (0.62, 7.94)	<0.001
TC, mmol/L (*n* = 1856)	4.4 (1.71, 19.65)	4.59 (1.71, 11.76)	4.32 (1.93, 19.65)	0.002
TG, mmol/L (*n* = 1856)	1.4 (0.31, 15.56)	1.5 (0.45, 13.94)	1.37 (0.31, 15.56)	0.027
SdLDL, mmol/L (*n* = 1856)	0.73 (0.04, 8.5)	0.82 (0.13, 2.31)	0.72 (0.04, 8.5)	<0.001
***Inflammation biomarker***, median (range)				
NLR (*n* = 1521)	3.25 (0.5, 48.78)	3.78 (0.8, 48.78)	3.15 (0.5, 43.14)	<0.001
HCY, umol/L (*n* = 1450)	12.60 (4.1, 76.3)	11.85 (5.8, 62.2)	12.70 (4.1, 76.3)	<0.001
CRP, mg/L (*n* = 614)	2.85 (0.0, 206.30)	2.20 (0.0, 206.3)	3.01 (0.0, 198.5)	0.715

Abbreviations: NIHSS: National Institutes of Health Stroke Scale; LDL: low density lipoprotein; TC: total cholesterol; TG: triglyceride; SdLDL: small and low-density lipoprotein cholesterol; NLR: neutrophil-to-lymphocyte ratio; HCY: homocysteine; CRP: C-reactive protein.

### APOE genotypes and stroke outcome

**Figure 2:**
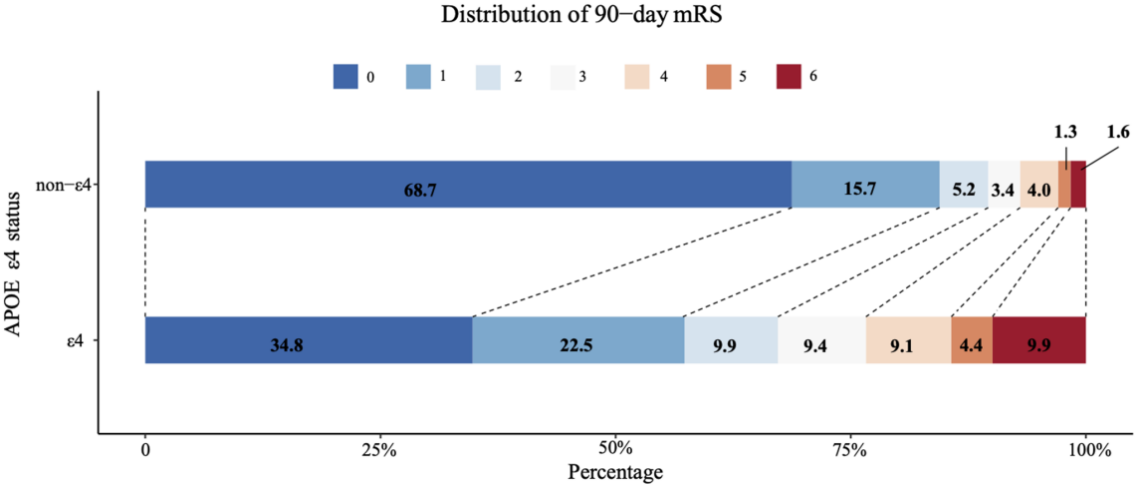
Comparison of 3-month mRS score between APOE ε4 carriers and APOE non-ε4 carrier.

At 3-month follow-up, 147 (42.98%) out of 342 ε4 carriers showed poor functional outcome (mRS ≥ 2), while 247 out of 1587 (15.56%) non-ε4 carriers have mRS ≥ 2. ε4 carriers seemed to have worse stroke outcome, as compared with non-ε4 carriers (mRS ≥ 2, 42.98% vs. 15.56%, *p* < 0.001; mRS ≥ 3, 32.75% vs. 10.40%, *p* < 0.001; death 9.94% vs. 1.64%, *p* < 0.001, Table 2, Figure 2).

**Table 2:** Association between APOE ε4 status and 3-month mRS score.

	**No. of events (%)**		**Crude analysis**		**Adjusted analysis**	
	**APOE ε4 noncarriers**	**APOE ε4 carriers**	**OR (95% CI)**	***P* values**	**OR (95% CI)**	***P* values**
mRS ≥2	247 (15.56)	147 (42.98)	4.09 (3.17–5.27)	<0.001	4.62 (3.51–6.09)	<0.001
mRS ≥3	165 (10.40)	112 (32.75)	4.20 (3.18–5.54)	<0.001	4.94 (3.63–6.72)	<0.001
Death (mRS = 6)	26 (1.64)	34 (9.94)	6.63 (3.92–11.20)	<0.001	8.35 (4.60–15.14)	<0.001

Note: The group of APOE non-ε4 carriers is reference. The multivariable logistic regression model adjusted for age (continuous), gender (female vs. male), smoking (yes, no or quit smoking), baseline NIHSS score (continuous), LDL level (continuous), atrial fibrillation (with or without), NLR (continuous), homocysteine (continuous). Abbreviations: OR: odds ratio; 95% CI: 95% confidence interval; mRS: modified Rankin scale; NIHSS: National Institutes of Health Stroke Scale; NLR: neutrophil-to-lymphocyte ratio; LDL: low density lipoprotein.

**Figure 3:**
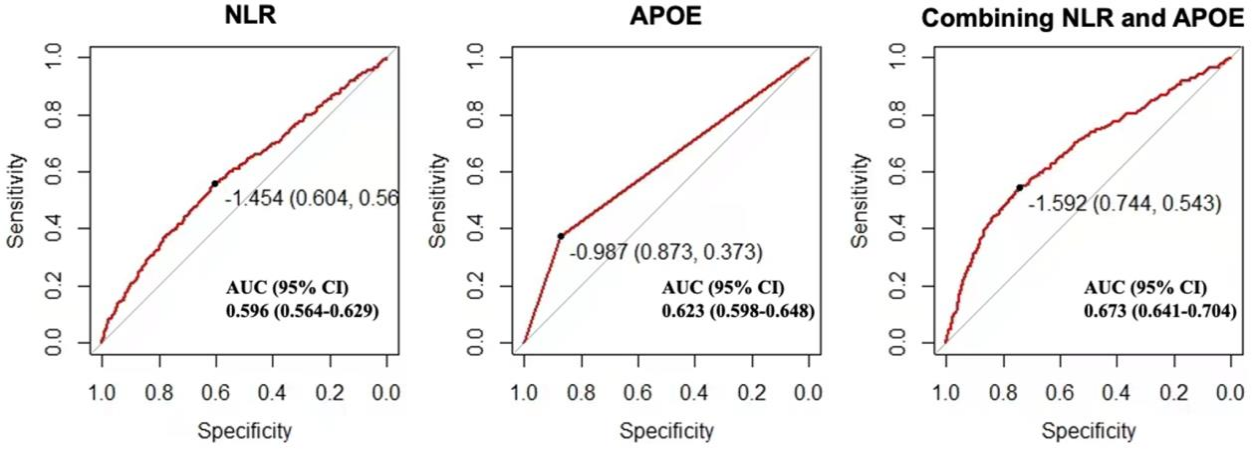
**Receiver operating characteristic curve for the prediction of 3-month stroke outcome in patients with acute ischemic stroke.** Predictive efficacy of APOE genotype and baseline neutrophil-to-lymphocyte ratio for 3-month mRS of 2–6. Abbreviations: NLR: neutrophil to lymphocyte ratio; CI: confidence interval.

As shown in Table 3, compared with APOE non-ε4 carriers, APOE ε4 carriers were strongly associated with poor functional outcome (OR = 4.62, 95% CI 3.51–6.09, *p* < 0.001) overall after adjustment for age, gender, smoking, baseline NIHSS score, LDL level, atrial fibrillation, NLR and homocysteine. The results were consistent in sensitivity analysis (Table 4). At the receiver operating characteristic (ROC) analysis, the area under the curve (AUC) of combining NLR and APOE genotype for poor functional outcome after ischemic stroke was 0.673 (0.641-0.704) (Figure 3.)

**Table 3:** Multivariable logistic regression analysis for unfavorable 3-month stroke outcome (mRS ≥2).

	**OR (95% CI)**	***P***
Age	1.03 (1.02–1.04)	<0.001
Gender, Female vs. Male	1.02(0.76–1.36)	0.916
Smoke		
Yes	Ref	
No	0.95 (0.69–1.31)	0.760
Quit smoking	0.79 (0.48–1.32)	0.377
APOE, ε4 vs. non-ε4 carriers	4.62 (3.51–6.09)	<0.001
NIHSS	1.11 (1.08–1.14)	<0.001
Atrial fibrillation	0.77 (0.50–1.18)	0.225
LDL level	0.89 (0.78–1.03)	0.109
NLR	1.02 (1.00–1.05)	0.089
Homocysteine	0.99 (0.97–1.01)	0.510

Abbreviations: NIHSS: National Institutes of Health Stroke Scale; NLR: neutrophil-to-lymphocyte ratio; LDL: low density lipoprotein; OR: odds ratio; 95% CI: 95% confidence interval.

**Table 4:** Odds ratios of APOE ε4 status for unfavorable 3-month stroke outcome.

**Multivariable-adjusted analyses**	**OR (95% CI), *p* value**	
	**mRS ≥2**	**mRS ≥3**
The main analysis^*^	4.62 (3.51–6.09), *p* < 0.001	4.94 (3.63–6.72), *p* < 0.001
Sensitivity analysis^†^	4.71 (3.56–6.24), *p* < 0.001	5.00 (3.66–6.82), *p* < 0.001
Sensitivity analysis^‡^	4.14 (2.44–7.03), *p* < 0.001	5.60 (3.13–10.02), *p* < 0.001

^*^Shown is the pooled results by using multivariable logistic regression model adjusting for age (continuous), gender (female vs. male), smoking (yes, no or quit smoking), baseline NIHSS score (continuous), LDL level (continuous), atrial fibrillation (with or without), NLR (continuous), homocysteine (continuous). All 1929 patients were included in the analysis. ^†^The analysis excluded the APOE ε4 homozygous carriers (*n* = 19) and repeated the logistic regression analysis with adjusting for the same covariates. ^‡^The analysis only included 614 patients with CRP information available and repeat the logistic regression analysis with adjustment for age, gender, smoking, baseline NIHSS score, LDL level, atrial fibrillation, NLR, CRP, and homocysteine. Abbreviations: mRS: modified Rankin scale; NIHSS: National Institutes of Health Stroke Scale; NLR: neutrophil-to-lymphocyte ratio; LDL: low density lipoprotein; CRP: C-reactive protein; OR: odds ratio; 95% CI: 95% confidence interval.

### APOE genotype and NLR

Table 5 shows the adjusted OR of unfavorable 3-month stroke outcome (mRS ≥ 2) for each sample-based quartile of NLR (i.e., Q2, Q3 Q4, vs. Q1). Although in the entire cohort, we did not observe an increased risk of 3-month unfavorable outcome (mRS ≥ 2) in patients with high quartile (Q2-4) of NLR, as compared with patients in Q1 (*p* for trend = 0.137), we observed differences in the relationship between NLR and unfavorable stroke outcome by APOE ε4 status. Among ε4 carriers, patients with high NLR had an increased risk of unfavorable stroke outcome relative to patients in low NLR (*p* for trend = 0.035). In contrast, among non-ε4 carriers, no significant difference in the risk of poor stroke outcome by NLR were found (*p* for trend = 0.772).

**Table 5:** Association between baseline NLR (quartiles) and 3-month mRS by APOE ε4 status.

**NLR level**	**Entire cohort *N* = 1521**		**APOE ε4 carriers *N* = 278**		**APOE non-ε4 carriers *N* = 1243**	
	**mRS ≥2 *n* (%)**	**OR (95% CI)**	**mRS ≥2 *n* (%)**	**OR (95% CI)**	**mRS ≥2 *n* (%)**	**OR (95% CI)**
Q1 (≤2.27)	68 (17.71)	REF	26 (35.14)	REF	42 (13.55)	REF
Q2 (2.28–3.28)	66 (17.41)	0.87 (0.59–1.28)	27 (38.03)	0.75 (0.35–1.60)	39 (12.66)	0.85 (0.53–1.44)
Q3 (3.29–5.00)	79 (20.90)	0.89 (0.61–1.31)	26 (46.43)	1.14 (0.51–2.53)	53 (16.46)	0.91 (0.57–1.44)
Q4 (5.01–48.78)	122 (32.11)	1.20 (0.83–1.75)	53 (68.83)	1.86 (0.85–4.09)	69 (22.77)	1.00 (0.62–1.60)
*P* values for trend		0.137		0.035		0.772

Note: All analyses were adjusted for age, gender and NIHSS scores. APOE ε4 carriers defined as ε4/ε4, ε4/ε3, and ε4/ε2. Abbreviations: OR: odds ratio; 95% CI: 95% confidence interval; Q: quartile; REF: reference.

## DISCUSSION

Our results demonstrate that APOE ε4 carriers who suffer AIS have worse functional outcome and higher mortality as compared with APOE non-ε4 carriers. Associations were also robust with adjustment for baseline NIHSS score suggesting that effects were mediated through mechanisms other than stroke burden. Among APOE ε4 carriers, we observed a significant association between NLR and poor functional outcome. However, the association was not found among APOE non-ε4 carriers.

Previous studies reported inconsistent results regarding association between APOE polymorphism and clinical outcome after ischemic stroke. Compared with previous studies, our study possesses several strengths. First, our analysis was based on a relatively large cohort of AIS patients. Furthermore, we included the lipid level and several inflammation index, which are closely related to the mechanism of APOE, into analysis. Our study found that APOE ε4 carriers suffered from worse functional outcome as compared with APOE non-ε4 carrier. However, we must recognize that genetic factors were not the only influence factor, and they may have interaction with other factors.

The reasons why APOE genotype influence stroke outcome remain understudied. It may be the results of multiple mechanisms. First, APOE genotype may modify the already strong risk factor for vessel disease to influence stroke recovery. In our study, patient with APOE ε4 have higher lipid level as compaired with non-ε4 carriers. Secondly, APOE genotype plays a critical role in determing the severity of the accumulation of amyloid-beta, which is associated with worser clinical outcome. Furthermore, APOE gene products were reported to be involved in inflammatory response and mitochondrial resistance to oxidative stress [22]. Finally, APOE ε4 may be directly linked with cerebrovascular dysfunction through mechanisms such as pericyte degeneration, endothelial cells alteration, smooth muscle cell damage [23].

Previous studies had found that NLR was an independent predictor of 3-month mortality after stroke [24, 25]. Among APOE ε4 carriers, we observed a strong and significant association between NLR and unfavorable functional outcome. However, the association did not exist among APOE non-ε4 carriers. The APOE genotypes-inflammation interaction have been reported in multiple other conditions, such as postoperative delirium [26], longevity [27] and Alzheimer’s disease [28]. Our study suggested that this interaction also exist in AIS patients. Although there are no definitive explanations for this relationship, one possible explanation may be that the more severe disruption of blood-brain barrier in APOE ε4 carriers favors the infiltration of peripheral immune cells into the site of injured tissue [29], which aggravates the inflammatory cascade. In addition, following stroke, the upregulation of vascular inflammation triggers pro-inflammatory marker adhesion to blood wall, which may engender AD-like cerebral damage [22]. This impact increases in APOE ε4 carriers and may increase infarct size, results in poor recovery after stroke [30].

Our finding that APOE genotype influence stroke outcome and has interaction with modifiable hematology index have clinical implications. First, health counselling that includes APOE genotype of patients may be reasonable, especially for those with high vascular risk. APOE ε4 carriers may need closer monitoring and much stronger control of risk factors of stroke. Furthermore, according to our findings, the combination of APOE genotype and baseline NLR at the onset of AIS may serve as a practical predictor of functional outcome in AIS. From a broader perspective, whether stroke patients can be benefit from inflammation prophylaxis may merit reconsideration and inclusion of genetic information. For example, NLR has been shown to be a marker associated with stroke-associated pneumonia and adverse clinical outcomes of cardiovascular disease [19, 31, 32]. Although infections may cause unfavorable functional outcome, the preventive antibiotics did not improve functional outcome in patients with acute stroke [33]. One possible explanation may be that those studies have not found the right candidates. Future studies may benefit from consideration of the added benefit of genetic factors, along with clinical variables in choosing the target patients for intervention.

Some limitations warrant mentioning as well. Given the retrospective nature of this study, some clinical information was not obtainable in part of our patients. Therefore, selection bias and potential confounding factors cannot be ignored. Whether our conclusion could be generalized to the whole ischemic stroke cohort needs further prospective study. Although our study provided the results of sensitivity analysis, we did not take all the inflammatory biochemical biomarker into consideration. It may not completely reflect the entire interaction.

In conclusion, the APOE ε4 carriers had worse functional outcome after AIS as compared with non-ε4 carriers. In addition, among APOE ε4 carriers, high NLR was associated with the increased risk of stroke-related disability, however, no such relationship was observed among APOE non-ε4 carriers. Combining the APOE genotypes and baseline NLR levels may be a practical predictor of functional outcome after AIS. Further prospective investigations with a larger cohort and sufficient information are needed to confirm these results.

## METHODS

### Patient cohort and eligibility

Consecutive patients with AIS within one week of onset admitted to the department of neurology at Sun Yat-Sen memorial hospital and First People’s Hospital of Foshan between January 2017 and March 2020 were screened. All patients would be followed up by trained research assistants who were blinded to patients’ APOE status. The severity of functional outcome was measured by mRS score which was assessed at 90 days through a standardized telephone interview [34]. We retrospectively collected the baseline demographic and clinical data of these patients through chart review. Patients whose major clinical data, APOE genotype, or 3-month mRS score were unobtainable were excluded. We also excluded those receiving reperfusion therapies (intravenous thrombolysis and/or endovascular thrombectomy) considering reperfusion therapy could greatly reduce disability. After application of our inclusion and exclusion criteria, 1929 patients were enrolled in our study (Figure 1).

### Baseline data collection

Ischemic stroke was defined using the WHO criteria. The following variables of interest were collected for each patient: age, gender, education, medical history (history of hypertension, history of diabetes mellitus, history of stroke), cigarette smoking status, alcohol drinking status, NIHSS score at admission, laboratory test results within 24 hours of admission to hospital, and 3-month mRS score. The ApoE genotype was determined using a commercial gene chip. NLR was calculated by dividing the absolute count of neutrophils by that of lymphocytes.

### Statistical analysis

Baseline categorical variables were reported as number of cases and percentages. Continuous variables conforming to normal distribution were expressed by means and standard deviations, while those not conforming to normal distribution were described by median (range). To consider the potential effects of APOE ε4 on the relationship between vascular risk factors and 3-month stroke outcome, we considered whether patients were ε4 carriers (i.e., ε4/ε4, ε4/ε3, ε4/ε2) versus non ε4 carriers. Chi-square test or analysis of variance (ANOVA) were used for comparison of baseline variables between groups with the different APOE genotypes. The primary outcome was dependence or death, defined as mRS of 2–6. Associations between APOE ε4 genotypes and 3-month mRS score were determined by comparing ε4 carriers with non-ε4 carriers. We used binary logistic regression to generate odds ratios (ORs) adjusted for age, gender, cigarette smoking status, history of atrial fibrillation, LDL level, inflammatory index (NLR and homocysteine), stroke severity (NIHSS) and also APOE ε4 genotypes. The sensitivity analyses were conducted in two ways: (1) to assess the influence of homozygous carriers of ε4, we excluded the APOE ε4 homozygous carriers and adjusted for the same covariates in the regression model; (2) we only included patients with CRP information available and repeat the regression analysis. The ability of the NLR and APOE genotype to predict the functional outcome was estimated through the receiver operatin characteristic (ROC) analysis. We performed tests for linear trend by entering the median value of each category of NLR as a continuous variable in the models.

All reported *p* values were 2-sided, with level of significance defined as *P* < 0.05. Statistical analysis was conducted using Stata 13.0 (Stata-Corp, College Station, TX, USA).

## Supplementary Materials

Supplementary Table 1
