# Supplementary material for: Association between Apolipoprotein E genotype and functional outcome in acute ischemic stroke
Source: Aging (Albany NY). 2023 Jan 13;15(1):108–18. doi: 10.18632/aging.204460 (PMC9876635; doi:10.18632/aging.204460)
Supplement: Supplementary Table 1 [file aging-15-204460-s001.pdf]

SUPPLEMENTARY TABLE

Supplementary Table 1. Baseline clinical characteristics between our study sample and the patients lost to follow-up.

Baseline characteristics	Our study group (*n* = 1929)	Patients lost to follow-up (*n* = 294)	*P* value
Age, mean/SD years	65.32/12.21	66.18/11.47	0.245
Male sex, *n* (%)	1277 (66.20)	187 (63.61)	0.382
Education > 9 years, *n* (%)	181 (9.38)	25 (8.50)	0.628
Atrial fabulation, *n* (%)	141 (7.31)	33 (11.2)	**0.020**
Hypertension, *n* (%)	1352 (70.09)	212 (72.11)	0.480
Diabetes mellitus, *n* (%)	575 (29.81)	89 (30.27)	0.871
Stroke history, *n* (%)	76 (4.03)	9 (3.79)	0.464
Smoke, *n* (%)	504 (26.13)	67 (22.79)	0.222
Drinking, *n* (%)	301 (15.60)	36 (12.24)	0.135
NIHSS score, median (range)	3 (0, 29)	3 (0, 24)	**0.001**
HbAlc, median (range)	5.9 (3.2, 15.9)	5.9 (3.5, 15.3)	0.704
Lipid level, median (range)			
Hdl, mmol/L	1.1 (0.08, 3.32)	1.1 (0.51, 2.09)	0.076
LDL, mmol/L	2.51 (0.62, 7.94)	2.53 (0.74, 9.06)	0.989
TC, mmol/L	4.4 (1.71, 19.65)	4.43 (1.92, 11.65)	0.571
TG, mmol/L	1.4 (0.31, 15.56)	1.28 (0.5, 10.16)	**0.045**
SdLDL, mmol/L	0.73 (0.04, 8.5)	0.68 (0.14, 2.22)	0.456
Inflammation biomarker, median (range)			
NLR	3.25 (0.5, 48.78)	3.76 (0.92, 37.78)	**0.001**
HCY, umol/L	12.6 (4.1, 76.3)	12.8 (4.7, 73.4)	0.987
**APOE ε4 carriers**	342 (17.73)	3(1.02)	<0.001

Abbreviations: NIHSS: National institutes of health stroke scale; LDL: low density lipoprotein; TC: total cholesterol; TG: triglyceride; SdLDL: small and low-density lipoprotein cholesterol; NLR: neutrophil-to-lymphocyte ratio; HCY: homocysteine.
